# Unusual Presentation of Hodgkin’s Lymphoma in Pregnancy: A Case Report and Systematic Review of Literature

**DOI:** 10.3390/hematolrep14040046

**Published:** 2022-11-03

**Authors:** Joseph Delzotto, Tahira. S. Naqvi, Nnennaya. U. Opara, Anthony Delzotto, Andrew Morgan

**Affiliations:** 1 Department of Emergency Medicine, Charleston Area Medical Center, Charleston, WV 25304, USA; 2 Department of Internal Medicine, Charleston Area Medical Center, Charleston, WV 25304, USA; 3 Department of Emergency Medicine, Charleston Area Medical Center Institute for Academic Medicine, Charleston, WV 25304, USA

**Keywords:** Hodgkin’s lymphoma, pregnancy, superior vena cava obstruction

## Abstract

Diseases occurring during pregnancy create a dilemma of managing the patient without causing harm to the unborn child. Three percent of the peak incidence of Hodgkin lymphoma (HL) is congruent with the reproductive period, particularly with pregnancy. Pregnant patients with HL always require a team of medical experts ranging from a medical oncologist, high-risk obstetrician, and neonatologist. Effective communication with both the patient and family is also necessary. The treatment goal for these patients should focus on achieving complete remission for the mother while permitting the delivery of a healthy child. Pregnant patients diagnosed with HL should undergo similar clinical investigations as other non-pregnant patients with accurate disease staging and appropriate non-radiation imaging such as ultrasound while avoiding invasive procedures.

## 1. Introduction

The simultaneous occurrence of cancer and pregnancy is an uncommon medical scenario and can pose a diagnostic and treatment challenge. Of malignancies diagnosed during pregnancy, hematologic malignancies rank second in the most prevalent neoplasms, after breast cancer, with Hodgkin’s lymphoma (HL) and non-Hodgkin’s lymphoma (NHL) comprising nearly 6% and 5%, respectively [1,2].

Classic Hodgkin’s lymphoma (CHL) comprises 95% of all HL cases, with subtypes characterized by clinical, phenotypic, genotypic, and morphological features. These subtypes include nodular sclerosing, mixed cellularity, lymphocyte depleted, and lymphocyte-rich [3]. A mediastinal mass occurs in most patients with nodular Sclerosing CHL. Clinical symptoms like fever, extreme fatigue, and night sweats can occur in about 25% of the cases [4]. In pregnancy, missed diagnosis is possible as lymphoma mimics the symptoms that typically accompany pregnancy, for example: dyspnea, fatigue, or night sweats. 

The management of lymphoma in a pregnant patient necessitates balancing the potentially harmful effects of diagnostics and therapeutic interventions on fetal development without compromising curative therapy for the mother.

We present a case of a patient presenting in the second trimester of pregnancy with superior vena cava (SVC) obstruction secondary to nodular sclerosing classical Hodgkin lymphoma NS-CHL, which was presumed to be a failure of treatment to a pneumonia outpatient.

## 2. Case Presentation

A 21-year-old female, 22 weeks pregnant Gravida 2 Para 0010 (G2P0010), was brought to the emergency department by the emergency medical services (EMS) for complaints of shortness of breath, facial swelling, lightheadedness, intermittent night sweats and one syncopal event. She denied any fevers or chills. She was treated outpatient for pneumonia with azithromycin and had no relief of symptoms. A week prior, the patient noticed that her breathing had become progressively worse after short walks, and she had noticed increased swelling in her legs and enlarged neck lymph nodes. Her examination revealed vital signs of temperature 98.9F, heart rate 113 beats per minutes, respiratory rate 26 breaths per minute, P0_2_ 99% on room air, and blood pressure of 99/70 mmHg. She had normal lung sounds, regular heart sound on (first heart sound S1 and second heart sound S2 on auscultation), and +1 pitting edema (up to 2 mm of depression which rebounds immediately upon release of pressure) in her right leg and cervical lymphadenopathy. The patient stated she had no significant past medical history and is not on any prescription medication except for prenatal vitamins. Patient is also a nonsmoker. Family history was unable to be obtained as the patient is adopted. She denied any previous surgical history. 

**Figure 1:**
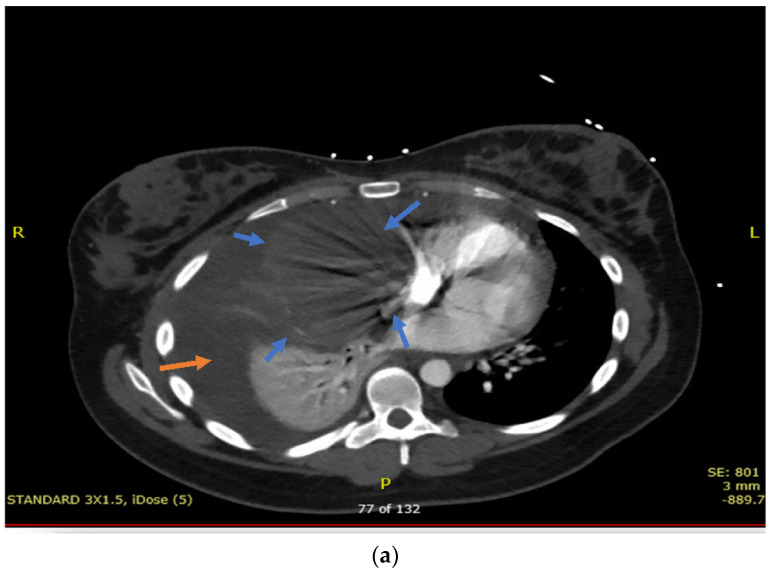

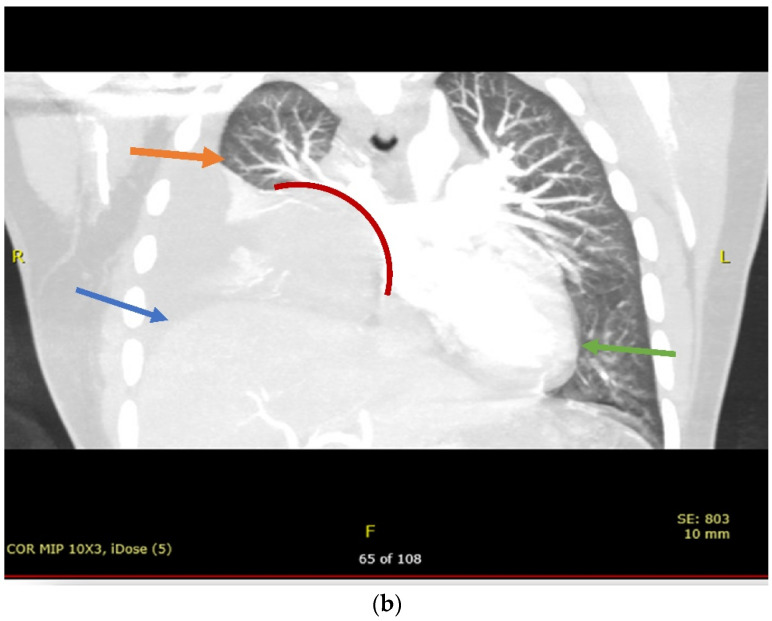
(**a**) Axial view of chest CTA showing mediastinal mass (blue arrows) compressing the right atria and ventricle along with right lung atelectasis and cardiac displacement to the left. Right sided pleural effusion (yellow arrow) can also be appreciated in the image. (**b**) Coronal view of chest CTA showing mediastinal mass compressing the right atria and right ventricle (red margin) along with right lung atelectasis (yellow arrow) and cardiac displacement to the left (green arrow). Right sided pleural effusion (blue arrow) can also be appreciated in the image.

Initial labs showed white count of 7.4 × 10^9^/L, hemoglobin of 10.3 g/dL, platelets 394 × 10^9^/L, creatinine 0.5 mg/dL, bilirubin 0.5 mmol/L, and lactate dehydrogenase (LDH) 269 U/L. Initially a chest X-ray was performed, which showed a large right sided consolidation with concerns for pleural effusion, which was concerning for pneumonia. A Computed tomography angiogram (CTA) of the chest was ordered to rule out a pulmonary embolism. However, the study detected a mediastinal mass right of midline measured 11.1 × 8.2 cm in size [Figure 1a,b]. 

Noted compression of the SVC with mass effect on the right atrium and ventricle with compressive atelectasis of the right middle and lower lobe was detected [Figure 1b]. 

Additionally, right-sided cervical lymphadenopathy was detected. A transthoracic echocardiogram showed a small pericardial effusion with a preserved ejection fraction. She was aggressively fluid resuscitated, with subsequent improvement in blood pressure. The patient was later admitted for further management with prompt consultation with cardiology, oncology, and obstetrics. 

**Figure 2:**
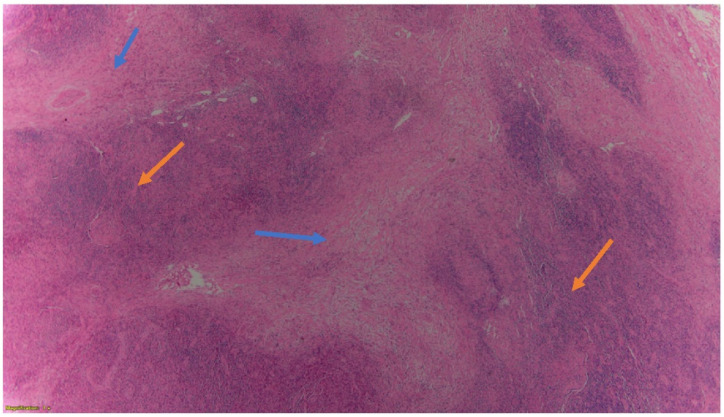
Hematoxylin and eosin magnified (4×) image of patient’s lymph node. Here, the tumor consists of nodules (yellow arrows) partly surrounded by collagen bands (blue arrows). The lymph node biopsy result also shows more than 80% of the nodules with fibrotic composition which is indicative of a grade II nodular sclerosing classical Hodgkin lymphoma (NS-CHL). Although these features do share similarities with anaplastic large cell lymphoma of the Hodgkin-like type (ALCL-HL), the later should only be considered only when the nodules consist exclusively of basophilic blasts. Thus, this differentiates our diagnosis from that of ALCL-HL.

**Figure 3:**
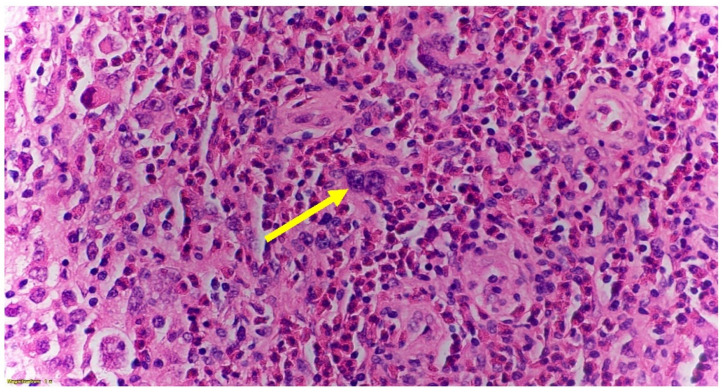
Magnified (60×) image of lymph node showing large structure with polylobular nuclei of a lacunar cell which are commonly seen in nodular sclerosing classical Hodgkin lymphoma NS-CHL (yellow arrow).

During the hospital course, the patient was evaluated by oncology, vascular surgery, ear nose throat (ENT) as well as Obstetrics. Vascular surgery recommended no immediate intervention for SVC compression. The patient had a biopsy of her right neck lymph node. The biopsy result showed fibrosis of the lymph nodes [Figure 2], and positive for classic Hodgkin’s lymphoma nodular sclerosing subtype with mixed cellularity grade 2 (based on the German lymphoma study group system and grade 1 by BNLI (British National Lymphoma Investigation) system) [Figure 3].

**Figure 4:**
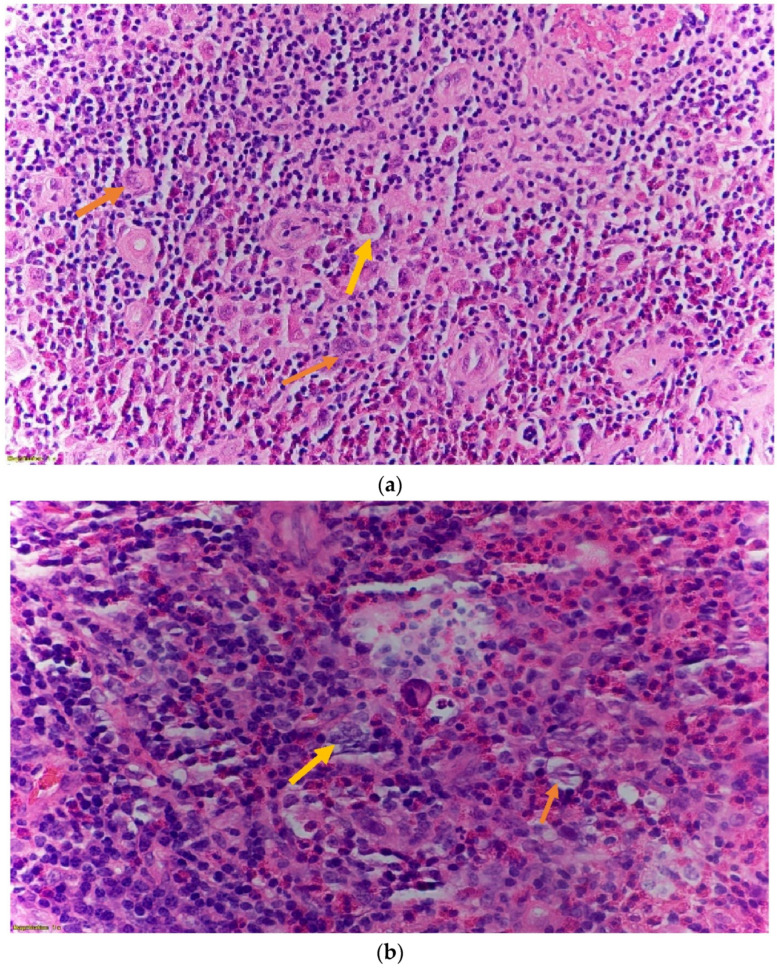
(**a**): lymph node microscopic slide magnified (40×) showing scattered lacunar cells (orange arrow) and abundant eosinophils in classical Hodgkin lymphoma. (**b**): H & E stain of a peripheral lymph node microscopic slide magnified (60×) showing numerous abnormal cells with bilobated nuclei, vesicular chromatin, prominent nucleoli, and abundant cytoplasm, indicative of mononuclear variants of Reed-Sternberg cell (yellow arrow).

**Figure 5:**
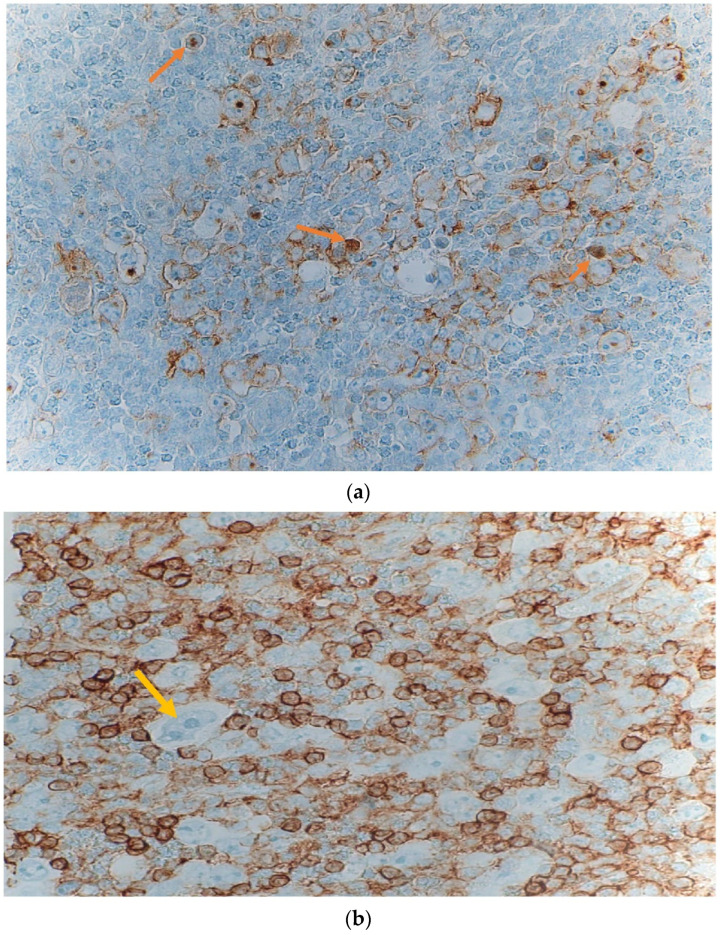
(**a**): Peripheral lymph node magnified (40×) Immunohistochemistry stain with CD30 showing weak nuclear staining (yellow arrows) greater than 10%. (**b**): peripheral lymph node magnified (60×) Immunohistochemistry with CD45 showing a “punched out” pattern with Lacunar cells and Reed-Sternberg cells with negative staining (yellow arrow).

A peripheral lymph node biopsy with histopathological slide further shows scattered lacunar cells, nodular mixed inflammatory infiltrate composed of prominent eosinophil predominance, and mild absolute lymphopenia with no blasts or atypical lymphocytes [Figure 4a and Figure 5a,b]. 

## 3. Methods Used for the Systematic Review of Literature

### 3.1. Search Criteria

A thorough database search on PubMed, EMBASE, and SCOPUS for published original articles and case reports describing the diagnosis, and management of Hodgkin lymphoma from year 1990 until August 2022 was performed. Using key word search. Each term was ‘Hodgkin lymphoma in pregnancy’, ‘Hodgkin lymphoma’, ‘HL in pregnancy’, ‘cancer in pregnancy’, and ‘pregnancy with HL’. The search strategy was limited to articles published in English language only. Boolean terms (AND, OR) were used to separate keywords. Medical Subject Headings (MeSH) were also used. The West Virginia university library was used to access all studies which were not open access.

### 3.2. Study Selection and Data Extraction

The selected articles were screened by N.U.O. The extracted articles were screened by their title, abstract and based on eligibility criteria. Duplicates were removed. The title and abstract of each study were screened and unrelated studies were excluded. The full texts of every article on Hodgkin lymphoma in pregnant women were included for synthesis. The data extracted include the first author, year of publication, chemotherapy adverse effects, HL subtype, treatment received, HL presenting symptoms, and fetal complications. Full text articles (review, original articles) and case reports were reviewed. Commentaries, Conference presentations, editorial, and short communication articles were also excluded.

### 3.3. Search Results

**Figure 6:**
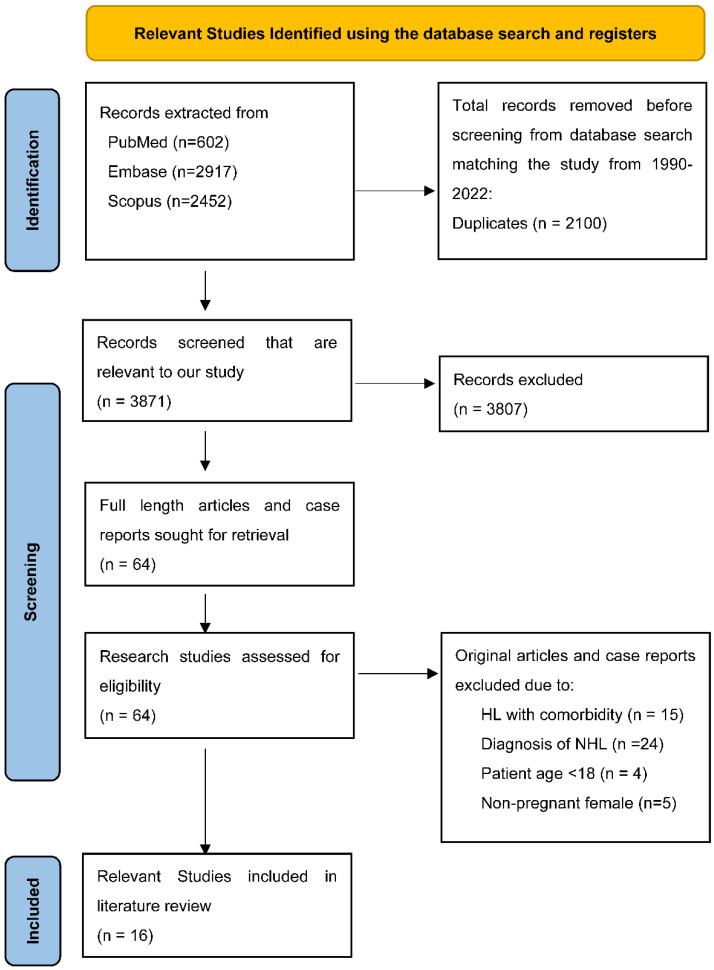
PRISMA 2020 flow diagram for new systematic reviews which included searches of databases and registers only.

Among 5971 studies retrieved from the three databases (602 from PubMed, 2917 from EMBASE, and 2452 from Scopus), a total of 2100 duplicate studies were removed, and 3871 studies were screened for titles and abstracts. Next, we excluded 3807 unrelated studies, and 64 studies were analyzed for full texts. Fourty-eight studies were later excluded based on the following reasons: 24 reported on non-HL patients, 4 reported HL in female patients below the age of 18, 5 studies reported on non-pregnant females with HL, and 15 studies reported on HL pregnant patients with comorbidity (immunocompromised). Additional searches from reference lists of the studies that were included found no other relevant studies. therefore, a total of 16 studies (4, 5, 6, 7, 8, 9, 10, 11, 12, 13, 14, 15, 16, 17, 18, 19) were added for further synthesis (Figure 6).

## 4. Results

### Characteristics of the Included Studies

The detailed characteristics of the included studies are depicted in Table 1. All studies were published between 1990–2022. All included studies were case reports and original articles. Ten studies diagnosed pregnant women with nodular sclerosis HL [4,5,6,7,8,9,10,11,12,13], Five described management of pregnant women diagnosed with lymphocyte-rich HL [14,15,16,17,18], and one study described management of pregnant patient with stage-4 classical HL [19]. Ten of the included studies treated all pregnant patients diagnosed with HL with standard cycle of ABVD with no report of maternal or fetal complications from the chemotherapy regimen [5,6,7,8,9,10,12,17,19], but with the exception of three studies; in which one of the three studies reported that ABVD chemo therapy resulted in preterm contraction and rupture of membrane [5], second study described the addition of 25 mg/m^2^ doxorubicin per cycle of ABVD which resulted in left cardiac dysfunction with high levels of troponin in the newborn on day 4 of life which later resolved on month one of life [16], and the third study reported an adverse effect with ABVD chemotherapy resulting in preterm birth, and the need for immediate post-partum blood transfusion with subsequent development of venous thromboembolism in the mother [11].

For the treatment of HL with R-CHOP (rituximab, cyclophosphamide, doxorubicin, vincristine, prednisolone), five studies [4,9,12,14,15] reported no complication with the chemo regimen in pregnant patients and their fetuses. It was observed that R-CHOP was commonly used for the chemotherapeutic regimen in pregnant patients diagnosed with lymphocyte-rich HL while ABVD regimen was more popular for the treatment of nodular sclerosing HL. However, one study [18] reported managing a pregnant patient diagnosed with HL with conservative/symptomatic treatment and postponing all chemo regimen until post-partum which did not result in worsening of patient’s or fetus’s health conditions [Table 1].

## 5. Treatment

The oncologist recommended treatment with ABVD (doxorubicin hydrochloride (Adriamycin), bleomycin sulfate, vinblastine sulfate, and dacarbazine) chemotherapy and continuation of chemotherapy throughout pregnancy, and radiation therapy to commence after delivery. Throughout her hospital course, the patient required oxygen support via nasal cannula. Due to the patient’s high risk for decompensation, she was transferred to another facility for advanced oncology management where she received two rounds of chemotherapy. Chest CT scans was avoided due to teratogenic concerns. Repeat chest X-ray showed resolution of the right sided pleural effusion. However, bilateral upper extremity duplex ultrasound showed a new right sided subclavian vein occlusion which was managed with Lovenox. The patient was eventually discharged home with outpatient follow-up with oncology. Six months later, a repeat chest CT scan showed decrease in mediastinal mass size, leftward heart displacement, and complete resolution of pericardial and pleural effusion. 

## 6. Pregnancy Outcome

The patient continued to be closely monitored throughout chemotherapeutic treatment. However, the attending obstetrician noted some fetal distress. A decision was made immediately on week 28 of pregnancy to deliver the neonate prematurely based on non-reassuring signs on fetal monitoring. The newborn was placed in the neonatal intensive care unit (NICU). There were no chemo-related malformation or adverse effects on the newborn. The patient’s newborn thrived in the NICU and was discharged without health complications. The mother continued with ABVD chemotherapy with radiation therapy during her post-natal period, with no other documented adverse effects.

## 7. Discussion

Lymphoma is the fourth most frequent malignancy in pregnancy, and Hodgkin lymphoma is diagnosed more frequently compared to non-Hodgkin lymphoma at the rate of 1 case of HL per 6000 pregnancies [13]. Accurately staging lymphoma is pivotal for appropriate management and determination of treatment plans. The primary aim of performing radiological imaging is to determine the staging of the cancer, for monitoring the disease progress, and to help determine if patient requires antenatal chemotherapy, if chemo can be delayed till postnatal period or both antenatal chemotherapy and postnatal chemotherapy treatment.

The decision to commence chemotherapy in pregnant patients depends on the severity of the patient’s presenting clinical signs and symptoms, including the gestation age during diagnosis, fetal risks regarding antenatal chemotherapy, and the potential severe consequences in delaying treatment. A multidisciplinary team of specialists are needed to develop a personalized treatment plan.

In limited studies, antenatal chemotherapy with standard regimens (non-antimetabolite) used for treatment at second or third trimester shows no increase in fetal morbidity or mortality [20,21,22]. Several treatment modalities, including immunotherapy and radiation chemotherapeutic, can be used; however, many of these treatment regimens is bespoke based on the biopsy results. The earlier the diagnosis, the more favorable the mortality outcome patients have. Those diagnosed with HL in pregnancy have 3-year survival rates of around 85% [23].

There has been no substantial evidence to support a compulsory use of chemotherapy regimen in all HL pregnant patients since chemotherapy could be delayed in some pregnant patients with little to no clinical symptoms. A review of current literature shows that ABVD is the treatment of choice particularly in HL (Grade 1C) when multi-agent chemotherapy is needed. Several research have shown that ABVD chemo regimen has an insignificant teratogenic effect when used at any stage of pregnancy, however, the reported cases are scant, and delayed toxicity in the child may be missed. Alkylating agents have been reported to have a very high teratogenicity in both animal and human studies, and so must be avoided during pregnancy particularly in the first trimester [24,25]. As a rule, chemotherapy or radiation therapy should be avoided during the first trimester of pregnancy as almost all chemo agents have been reportedly teratogenic in both animal and human studies. Some chemotherapeutic drugs when used in first trimester of pregnancy have resulted in spontaneous abortion, major fetal malformation, and fetal death. This is based on fetus vulnerability during the first trimester when organogenesis occurs, and post organogenesis period (2–8 weeks gestation), several organs including the genitalia, eyes, hematopoietic organs, and the central nervous system, continue to be vulnerable to radiation and chemo toxicity. When clinical symptoms of Hodgkin lymphoma become life-threatening in the first trimester (although rare), patient is often advised to terminate the pregnancy.

Most pregnant patients diagnosed with HL are often seen at the stages 1 and 2 and are either asymptomatic or have mild symptoms. Pregnant patients diagnosed with HL with no clinical symptoms are often monitored closely and treatment deferred till after childbirth [26]. The idea of watchful waiting among these patients have been studied among 19 pregnant patients with HL which proved to be successful and confirms that many pregnant patients with HL and with no clinical symptoms can be monitored throughout pregnancy until childbirth without chemotherapy for the lymphoma [27].

The management of pregnant patient with advanced HL or HL with severe B-symptoms have proven to be successful especially with the ABVD combo. Which is the current standard of care in North America. Studies have shown that children born to women who received ABVD chemo regimen for HL in the second and third trimester delivered healthy newborns with no secondary malignancies or defect [28]. Following childbirth, the post-natal oncologic care is a very important step in managing HL in pregnancy. The timing and delivery of the baby in HL pregnant patients require team members (oncologist and obstetrician) to plan the timing and mode of delivery. The pregnancy stage and fetal maturity should guide the criterion for labor induction. The induction of labor should be aimed between 36 weeks and 37 weeks. After the delivery of the newborn, the placenta pathology examination should be performed to rule out metastasis [29]. Studies have also shown that 54% of neonates exposed to chemotherapy in their second and third trimester were born preterm, with high rate of neonatal intensive care unit (NICU) admission. However, approximately 90% of most of the births were due to induction of labor, and the need for NICU were due to pre-maturity [1,30]. Therefore, preterm delivery should be avoided, if possible, even though it was not the case in our patient due to fetal distress from chemo treatment resulting in preterm birth. Furthermore, at post-natal period, a team of specialists comprising of neonatologist, obstetrician with High-risk pregnancy experience, and oncologists should create a peripartum plan for these patients to minimize complications. Patients who require continuous chemotherapy must avoid breastfeeding as most chemo drugs can be excreted into breast milk. Patients who received chemotherapy during pregnancy for HL should be fully restaged at post-natal period with PET scan or CT scan, and these scans can also be used to assess the depth of post-therapy remission.

The survival of pregnant patients diagnosed with classical HL was compared with that of non-pregnant women treated for HL in a study documented in the international Network on Cancer registry, comprising of 134 pregnant patients with HL [5]. The two groups had same stage and prognostic scores at diagnosis and were treated with ABVD chemo regimen. The result showed that 120 (90%) of the patients in the two groups delivered healthy neonates, and 47 patients had preterm delivery. The 5-year survival rate for mothers with early-stage HL vs. advanced HL were 97.3% and 100%, respectively, with 5-year cancer-free for mothers with early-stage HL vs. advanced HL at 83% vs. 91%, respectively, [5]. 

Treatment of HL in pregnancy are somewhat similar to HL treatment regimen in non-pregnant women. However, in non-pregnant women, ABVD and escalated BEACOPP (bleomycin, etoposide, doxorubicin, cyclophosphamide, vincristine, procarbazine, prednisone) are the most common chemotherapeutic regimen for HL [31]. A case–control study of 36 non-pregnant women treated for HL with ABVD and who attempted pregnancy were successful, and no evidence of significant fertility impairment, and ABVD is not associated with risk of premature menopause [32]. However, the German Hodgkin study Group (GHSG) analyzed the menstrual status of 405 females treated for HL with eight cycles of BEACOPP [33]. The result showed that 51.4% of the patients who were treated with BEACOPP developed permanent amenorrhea. The higher rate of amenorrhea was associated with escalated BEACOPP regimen than standard BEACOPP. Another study [34] comprising of 518 females treated for HL and were followed for a period of 9.4 years, 97 women developed premature menopause before the age of 40. The study also showed that chemotherapy linked to a 12.3-fold increase in premature menopause when compared to HL treatment with radiotherapy alone. on the other hand, abdominal and pelvic radiotherapy increases the risk of premature ovarian failure.

## 8. Conclusions

HL in pregnancy and its treatment is a relatively understudied area of medicine due to its many ethical boundaries and complications of treatment options. This case gives us insight into challenging and difficult decisions made with shared decision-making between patient and physician. Studies suggest that HL in pregnancy shows promising results with ABVD as a regimen of choice if multi-agent chemo is required. Alkylating agents should be avoided in the first trimester due to teratogenic effects. A broad multidisciplinary team-based approach is vital for improved patient outcomes. Additional research and a registry of children born to treated HL patients are necessary.

## Figures and Tables

**Table 1:** Studies showing clinical symptoms, chemotherapeutic regimen, chemo adverse effects, and fetal complications in pregnant patients diagnosed with Hodgkin lymphoma.

Author/Year	Chemo Side Effects	HL Diagnosed Subtype	Treatment Received	HL Presenting Symptoms	Fetal Complication
Buchholz, M. L. et al., 2018 [4]	Fever	Nodular sclerosing	R-CHOEP (rituximab, cyclophosphamide, doxorubicin, vincristine, etoposide, prednisolone)	Dry cough, dyspnea, facial edema with hyperemia, night sweats, hepatosplenomegaly	None
Maggen, C. et al., 2019 [5]	Preterm contractions or preterm rupture of membrane	Nodular sclerosing	Standard ABVD (doxorubicin, bleomycin, vinblastine, dacarbazone)	Dry cough, dyspnea, night sweats	None
De Sanctis, V. et al., 2012 [6]	Fever	Nodular sclerosing	ABVD (Doxorubucin hydrochloride (Adriamycin), Bleomycin sulfate, Vinblastin sulfate, and Dacarbazin	Dry cough, night sweats, fatigue	None
Aviles, A. et al., 2018 [7]	Fever	Nodular sclerosing	ABVD (Doxorubucin hydrochloride, Bleomycin sulfate, Vinblastin sulfate, and Dacarbazin	Fatigue, Night sweats, dyspnea	None
Dunleavy, K. et al., 2020 [14]	Fever	Lymphocyte -rich	Total of 6-cycle of R-CHOP (Rituximab, cyclophosphamide, doxorubicin hydrochloride, Vincristine (Oncovin), Prednisone)	Chest pain, dyspnea, cramps in right calf	None
Bachanova, V. et al., 2013 [15]	Fever	Lymphocyte -rich	R-CHOP (Ritoximab, Cyclophosphamide, Doxorubicin hydrochloride, Vincristine (Oncovin), Prednisone)	Dyspnea, night sweats, dry cough	None
Eyre, T. A. et al., 2015 [8]	Fever, fatigue	Nodular sclerosing	ABVD (Doxorubucin hydrochloride, Bleomycin sulfate, Vinblastin sulfate, Dacarbazin) with single vinblastine in first trimester	Dyspnea, night sweats, fatigue	None
Gurevich-Shapiro, A. et al., 2019 [9]	Fatigue, fever, anorexia	Nodular sclerosing	R-CHOP (Ritoximab, Cyclophosphamide, Doxorubicin hydrochloride, Vincristine (Oncovin), Prdniisone)	Night sweats, fatigue, dyspnea, dry cough	None
Cotteret, C. et al., 2020 [16]	Fatigue, fever	Lymphocyte-rich	2-cycle Standard ABVD (Doxorubucin hydrochloride, Bleomycin sulfate, Vinblastine sulfate, Dacarbazin) with 25 mg/m^2^ doxorubicin per cycle	Night sweats, dry cough, fatigue, dyspnea	Left cardiac dysfunction on day-4 after birth with high level of troponin which later resolved at 1 month of life.
Moshe, Y. et al., 2017 [10]	Fever, anorexia, nausea	Nodular sclerosing	ABVD (Doxorubucin hydrochloride, Bleomycin sulfate, Vinblastine sulfate, Dacarbazin)	Dry cough, fatigue, dyspnea, night sweats	None
El-Messidi, A. et al., 2015 [11]	Preterm birth, needed immediate post-partum blood transfusion, venous thromboembolism	Nodular sclerosing	ABVD (Doxorubucin hydrochloride, Bleomycin sulfate, Vinblastine sulfate, Dacarbazin)	Dry cough, night sweats, fatigue	None
Aisner, J. et al., 1993 [12]	None	Nodular sclerosing	R-CHOP (Ritoximab, Cyclophosphamide, Doxorubicin hydrochloridee, Vincristin (Oncovin), Prednisone	Night sweats, fatigue	None
Iriyama, N. et al., 2011 [19]	None	Stage-4 classical HL	Total of 6-cycle of ABVD (Doxorubucin hydrochloride, Bleomycin sulfate, Vinblastine sulfate, Dacarbazin)	Dry cough, fatigue, night sweats	None
Lishner, M. et al., 1992 [17]	None	Lymphocyte-rich	ABVD (Doxorubucin hydrochloride (Adriamycin), Bleomycin, Vinblastine sulfate, Dacarbazin)	Night sweats, dry cough, dyspnea	None
Anselmo, A. P et al., 1999 [18]	Anorexia, fever	Lymphocyte-rich	ABVD (Doxorubucin hydrochlorid (Adriamycin), Bleomycin sulfate, Vinblastine sulfate, Dacarbazin)	Dry cough, night sweats, weight loss, dyspnea	None
Korkontzelos, I. et al., 2005 [13]	None	Nodular sclerosing	Conservative/symptomatic treatment. Chemotherapy was given post-natal.	Dry cough, night sweats, fatigue	None

## Data Availability

The data used in this study was accurately cited within the manuscript. Further inquiries can be directed to the corresponding author.
